# 31 Barriers to Physical Activity in Pregnant Women: An Explanatory Sequential Mixed Method Study

**DOI:** 10.1093/eurpub/ckae114.152

**Published:** 2024-09-26

**Authors:** Atefeh Omrani, Romina Fili, Sana Nazmi, Fereshteh Behmanesh, Hossein-Ali Nikbakht, Leila Amiri Farahani

**Affiliations:** University Of Sunderland In London, London, United Kingdom; Babol University of Medical Sciences, Babol, Iran; Babol University of Medical Sciences, Babol, Iran; Babol University of Medical Sciences, Babol, Iran; Babol University of Medical Sciences, Babol, Iran; Iran University of Medical Sciences, Tehran, Iran

## Abstract

**Purpose:**

Regular physical activity during pregnancy has many benefits including reducing the risk of preeclampsia, gestational diabetes, improving physical fitness and reducing the risk of excessive maternal weight gain. However, pregnant women often choose a more sedentary lifestyle, driven by false misconceptions and beliefs (Coll et al., 2017). Gaining insights into the obstacles that hinder women from engaging in physical activity during pregnancy is crucial for guiding future interventions aimed at enhancing physical activity levels. The research aims to identify barriers to physical activity among pregnant women using an explanatory sequential mixed-methods approach.

**Methods:**

This research employs an explanatory sequential mixed-methods design. The project will be implemented in two separate phases. In the first phase, a quantitative cross-sectional study will be conducted with 358 eligible pregnant women aged 18-45 years in collaborations with researchers in Iran and the UK. The collection of quantitative data will involve the use of the Barriers to Physical Activity during Pregnancy Scale (BPAPS) along with questionnaires gathering demographic and obstetric data. Following the analysis of the quantitative data and the identification of qualitative objectives, the study will progress to its second phase. During this stage, a qualitative investigation will take place, employing individual semi-structured interviews utilizing a content analysis approach.

**Results:**

This is an ongoing study (Fili et al., 2024). Initially, the mean score of BPAPS and its subscales, along with the correlation between demographic and obstetric variables and the mean score of BPAPS, will be reported. Subsequently, the qualitative phase will encompass the reporting of categories and the main themes. Finally, the interpretation of the quantitative phase findings will be detailed, incorporating insights gained from the qualitative phase.

**Conclusions:**

Since regular physical activity during pregnancy has many benefits for maternal and fetal health, the findings of this study can play a vital role in strategic planning to address women’s false beliefs and misconceptions regarding physical activity during pregnancy. In addition, this study will contribute to designing interventions to remove barriers to physical activity, encouraging physical activity participation in pregnant women, hence improving the overall health and wellbeing of women, especially during pregnancy.

